# A new technique inversion Time-Domain electromagnetic data

**DOI:** 10.1016/j.heliyon.2023.e21638

**Published:** 2023-11-04

**Authors:** Widodo

**Affiliations:** a Applied Geophysics and Exploration Group, Geophysical Engineering, Faculty of Mining and Petroleum Engineering, Bandung Institute of Technology, Indonesia

**Keywords:** Inversion, Biology, mSOS, TDEM

## Abstract

Time-Domain Electromagnetic (TDEM) data modeling, especially for central-loop configurations, is often achieved through 1D inversion models. This study aims to enhance the accuracy and efficiency of TDEM data inversion by employing the Born Approximation method to address calculation and convergence speed issues. We also utilize the modified Symbiotic Organism Search (mSOS), a global optimization algorithm capable of handling multi-minimum problems in non-linear objective functions, to optimize the inversion process. Our research includes the assessment of the accuracy and performance of this approach through inversion modeling on both synthetic and field data. The accuracy of the synthetic data was evaluated based on the algorithm's capability to retrieve the values of the synthetic data, as indicated by the small relative error between the synthetic model parameters and the calculated model. In the case of field data modeling, the accuracy relied on the consistency achieved when modeling the data with different numbers of layers. Additionally, we considered the time required to perform the inversion as an evaluation metric for inversion performance. For the synthetic data case, the algorithm produced relatively accurate models with misfit values of approximately 0 % and low relative error values. In the field data case, the inversion models demonstrated consistency and reduced misfit values when the data was modeled with different numbers of layers, specifically 8.72 % for the 2-layer model, 3.92 % for the 3-layer model, and 2.61 % for the 4- and 5-layer models. Both datasets required less than 19 min for 10,000 iterations. These findings highlight the innovative nature of the mSOS algorithm and its potential for practical applications in TDEM inversion studies.

## 1 Introduction

In recent years, there has been a surge in the popularity of derivative-free nature-inspired metaheuristic algorithms across various applications. These algorithms, which do not rely on explicit derivative information, offer a powerful and versatile approach for solving complex optimization problems in diverse fields. Derivative-free nature-inspired metaheuristic algorithms draw inspiration from natural processes and phenomena, such as the behavior of organisms, evolutionary principles, or swarm intelligence. These algorithms exhibit the ability to explore large solution spaces, handle nonlinearity, and find optimal or near-optimal solutions even in the absence of gradient information. There are several global optimization algorithms that have been implemented to conduct inversion geophysical data. The improvement of the Modified Symbiotic Organism Search (mSOS) algorithm has been applied for Self Potential and Vertical Electrical Sounding data [1]. The application of Differential Search Algorithm (DSA) gives promising better parameters yielded for inversion of Horizontal Loop Electromagnetic (HLEM) data [2].

The principal aim of this study is to seamlessly integrate the innovative mSOS algorithm, acknowledged as a cutting-edge global optimization technique. The primary focus is devoted to unlocking the complete potential of mSOS in the complex undertaking of 1D inversion modeling of Central-loop Time Domain Electromagnetic (TDEM) data. This effort is poised to propel the frontiers of optimization methodologies, thereby enriching the landscape of scientific exploration in the field.. The inversion of TDEM data has been extensively studied, and various global optimization algorithms have been employed for this purpose, including the Genetic Algorithm (GA) [3], Particle Swarm Optimization (PSO) [3], Firefly Algorithm (FA) [4], Born Approximation, and Levenberg-Marquardt Algorithm [5], as well as a combination of Flower Pollination Algorithms (FPA) and elitism techniques [6].

Geophysical data, including TDEM data, typically exhibit a non-linear correlation between their parameters. This non-linearity makes the inversion process challenging and requires the application of advanced optimization techniques. Global optimization algorithms offer convenient procedures for generating initial models for the inversion of geophysical data by randomly producing starting models within predetermined parameter value intervals. Moreover, one of the key advantages of using global optimization algorithms is their ability to produce global optimum solutions rather than local minimum solutions, which is particularly beneficial when dealing with objective functions that may have multiple minima.

In this study, we implemented the Born Approximation, developed by Christensen [7], to perform forward modeling and generate the TDEM response. The Born Approximation is widely used in electromagnetic geophysical studies and provides a mathematical framework for modeling the response of subsurface structures to electromagnetic fields. By applying the Born Approximation, we can simulate the TDEM response based on a given subsurface model and compare it with the observed data.

To assess the mSOS algorithm's capabilities, we conduct experiments using both synthetic and field data. Synthetic data enables controlled experiments and serves as a known reference model for evaluating inversion results. We consider various criteria, including the misfit value between observed and calculated data, inversion time, and relative error between synthetic and calculated models.

Field data, representing real-world scenarios, adds complexity and uncertainty due to the inherent characteristics of subsurface structures. By analyzing the mSOS algorithm's performance on field data, we can evaluate its practical applicability and reliability in addressing real-world geophysical inversion challenges.

All forward modeling and inversion procedures are implemented using MATLAB 2021, a versatile numerical computing environment suitable for data analysis and optimization. In summary, this research leverages the mSOS algorithm for 1D TDEM data inversion, emphasizing the advantages of global optimization algorithms in geophysical inversion. The Born Approximation facilitates forward modeling, and experiments with synthetic and field data assess the algorithm's performance in terms of misfit value, inversion time, and consistency. The results obtained using MATLAB 2021 serve as the foundation for evaluating the proposed approach's effectiveness and practicality.

## 2 Method

### 2.1 Forward modeling

In the Central Loop TDEM method, an artificial source induces a current in the subsurface due to the magnetic field variation over time ($\frac{\partial H_{z}}{\partial t}$). This leads to the generation of secondary magnetic fields and secondary derivative magnetic fields, which are measured during data acquisition [8]. The derivative of the secondary magnetic field for a homogeneous half-space model can be calculated using equation (1) provided below:

(1) $$\frac{\partial H_{z}}{\partial t}=-\frac{I}{\mu_{0}\sigma a^{3}}\left[3\;\mathrm{erf}\left(\theta a\right)-\frac{2}{\sqrt{\pi }}\left(3+2\theta^{2}a^{2}\right)e^{-\theta^{2}a^{2}}\right]$$

θ and the error function (erf(θa)) associated with this calculation is determined by equation (2), (3) mentioned below:

(2) $$\theta =\sqrt{\frac{\mu_{0}\sigma }{4t}}$$

(3) $$\mathrm{erf}\left(\theta a\right)=\frac{1}{\sqrt{\pi }}\int_{-\theta a}^{\theta a}e^{{-t}^{2}}dt$$

For preliminary or quantitative interpretation of the subsurface before conducting inversion modeling, the secondary derivative magnetic field can be transformed into late-time apparent resistivity ($\rho_{a}$) using equation (4) given as follows:

(4) $$\rho_{a}\approx \sqrt[{3}]{\frac{I^{2}{\left(\mu_{0}\right)}^{3}a^{4}}{400\pi t^{5}}{\left(\frac{-\partial H_{z}}{\partial t}\right)}^{-2}}$$

To calculate the TDEM data response as a forward modeling method, we implemented the Adaptive Born Forward Mapping. The relationship between the TDEM data and the parameter models in the subsurface is connected by the apparent conductivity for each layer as a function of the measurement time with index j. The apparent conductivity ($\sigma_{a}$) can be calculated using equation (5) provided below:

(5) $$\sigma_{a}\left(t_{j}\right)=\sum_{k=1}^{L}\sigma_{k}F_{jk}$$

$\sigma_{k}$ is a synthetic conductivity model for each layer with index *k*. The transformation of synthetic conductivity models became apparent conductivity was accommodated by integral of Fréchet kernel $\left(F_{jk}\right)$ in dependence upon depth (*z*), the integral of Fréchet kernel calculated by linear approximation which can be computed by this following equation (6).

(6) $$F\left(z_{k},t_{j},\sigma_{a}\left(t_{j}\right)\right)=f\left(x\right)=\left\{ \begin{array}{c} \frac{z_{k}}{D_{j}}\left(2-\frac{z_{k}}{D_{j}}\right),{for\;z}_{k}\leq D_{j}\\ 1,{for\;z}_{k}>D_{j} \end{array}\right.$$

Variable $D_{j}$ determined this following equation (7).

(7) $$D_{j}=\sqrt{\frac{ct_{j}}{\mu_{0}\sigma_{a}\left(t_{j}\right)}}$$

In this research, the value of the constantan c is set to 1.2, which affects the sensitivity of the Fréchet kernel. The calculation of apparent conductivity undergoes an iterative process to update the TDEM data response value until it reaches the minimum change of the TDEM data response value for each iteration. The update process is conducted using the following equation (8):

(8) $$\sigma_{a}^{k}=\alpha \sigma_{a}^{k+1}+\left(1+\alpha \right)\sigma_{a}^{k}$$

The iteration process requires initialization $\sigma_{a}^{k}$, which is calculated as the average of the conductivity model. In this research, the number of iterations and are set to 10 and 0.4, respectively, based on experimental observations to achieve the minimum change of the TDEM data response value for each iteration.

### 2.2 Inversion modeling

The mSOS algorithm is a global optimization algorithm inspired by symbiotic relationships found in nature, including mutualism, commensalism, and parasitism [9,10]. Mutualism describes a relationship between two organisms that benefit each other. These types of relationships are translated into mathematical equations that exhibit similar characteristics to their original definitions. In the mutualism stage of the algorithm, initialization is performed by generating several random parameter models. For the inversion problem in 1D TDEM data, the parameter model consists of two parameters: resistivity and thickness of the lithology.

To create random parameter models, priori information in the form of range values, with lower and upper boundaries for each parameter, needs to be determined at the beginning. In the context of TDEM data inversion, the lower boundary of resistivity is denoted as ($\rho_{\min})$, the upper boundary of resistivity as ($\rho_{\max}$), the lower boundary of thickness as ($h_{\min}$), and the upper boundary of thickness as ($h_{\max}$). This range of values is referred to as the search space. The number of boundaries for each parameter model depends on the number of layers (nlayers) in the model. In a 1D model, there is a concept of a homogeneous half-space, where the last layer is assumed to have infinite depth. Therefore, the number of parameter models for resistivity is the same as the number of layers ($n_{layer})$, while the number of parameter models for thickness is $\left(n_{layer}-1\right)$. Hence, the total number of parameters for the 1D model is $\left({2x\;n}_{layer}-1\right)$, which simplifies to $\left({2x\;n}_{layer}-1\right)$. Random parameter models can be generated using the following equation (9), (10):

(9) $$\rho_{i,j}=\rho_{\min}+Rand\left(0,1\right)\;x\;\left(\rho_{\max}-\rho_{\min}\right)$$

(10) $$h_{i,j}=h_{\min}+Rand\left(0,1\right)\;x\;\left(h_{\max}-h_{\min}\right)$$

**Fig. 1:**
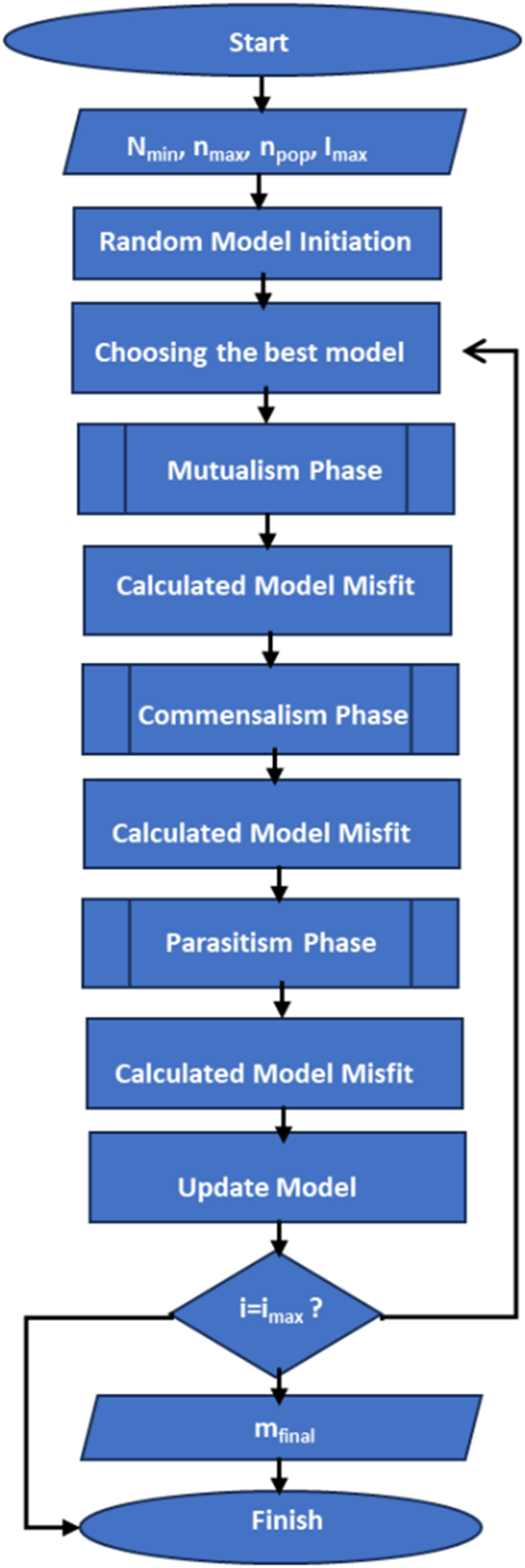
Flowchart of inversion of central loop TDEM data using mSOS algorithm.

The random number “Rand (0,1)" represents a random value within the interval of 0–1. The variables “i" and “j" serve as indices for each organism and parameter model, respectively. In the next stage, the random parameter models will be evaluated by calculating the TDEM response and misfit value. Among these models, the best organism representing the model with the smallest misfit value will be selected. Subsequently, an iterative process involving mutualism, commensalism, and parasitism stages will take place to generate new candidate solutions. This process includes comparing the new candidate solution with the old candidate solution to determine which one is superior based on the misfit value. Afterward, the candidate solutions will undergo elimination to obtain the optimal model during each iteration. To provide a brief overview of the inversion process, a flowchart is presented in Fig. 1. The subsequent subsection will provide a detailed explanation of each step.

#### 2.2.1 Mutualism stage

In the mutualism stage, two organisms, represented by $X_{j}$ and $X_{k}$, are selected with the condition $X_{i}$ ≠ $X_{j}$ ≠ $X_{k}$. $X_{k}$ is randomly chosen from the ecosystem and serves as a model for $X_{i}$ and $X_{j}$ to converge towards. The relationship between $X_{i}$ and $X_{j}$ is represent by mutual vector ($X_{mut}$ that can be calculated with averaging the value of $X_{i}$ and $X_{j}$. The Benefit Factor (BF) is set to 1. The new candidate solution in this stage is determined using the following equation (11), (12), (13):

(11) $$X_{i,new}=X_{i}+Rand\left(0,1\right)\;x\;\left(X_{k}-X_{mut}x\;BF\right)$$

(12) $$X_{j,new}=X_{j}+Rand\left(0,1\right)\;x\;\left(X_{k}-X_{mut}\;x\;BF\right)$$

(13) $$X_{mut}=\frac{X_{i}+X_{j}}{2}$$

*Rand (0, 1)* is a random number that has interval value between 0 up to 1. The new candidate solution in mutualism stage will be evaluated and compared with the old solutions of misfit value. If the new solution has a lower misfit than the old solution, the new solution will replace the old solution. Contrariwise, if the old solution has a lower misfit than the new solution, the old solution will be kept as a candidate solution. The solution which is obtained in this stage will continue to the next stage.

#### 2.2.2 Commensalism stage

In commensalism stage algorithm will improve the exploitation capability to search a solution in local area that will be impact to speed of convergency. One organism ($X_{j}$) will be elected randomly in the ecosystem with condition $X_{i}$ ≠ $X_{j}$. $X_{best}$ as organism that has smallest misfit values among all organisms will be pick out as a reference for $X_{j}$. The new candidate solutions ($X_{i,new}$) for this stage calculated by this following equation (14).

(14) $$X_{i,new}=X_{i}+Rand\left(0.4,0.9\right)\;x\;\left(X_{best}-X_{j}\right)$$

*Rand (0.4, 0.9)* is a random number that has a range value between 0.4 until 0.9. The new solution in this stage will be evaluated and compared with the old solutions considering by misfit value. If the new solution has a lower misfit than the old solution, the new solution will replace the old solution and vice versa. The candidate solution that obtained in this stage will continue to the next stage.

#### 2.2.3 Parasitism stage

In the parasitism stage, the algorithm aims to increase its ability to explore solutions in the global area. One random organism ($X_{j}$) with conditions $X_{i}$ ≠ $X_{j}$. $X_{i}$ is selected from the ecosystem with the condition. $X_{p}$ is then changed to produce the parasite vector using the following equation (15):

forj≠Rand(1,M)

others

(15) $$X_{p}=\left\{ \begin{array}{c} X_{i,j}\\ X_{\min}+Rand\left(0,1\right)\;x\;\left(X_{\max}-X_{\min}\right) \end{array}\right.$$

Here, Rand(1, M) is a random number between 1 and the number of parameters (M). The parasite vector can replace as the new candidate solution after comparing the two based on their misfit values. If the misfit value of $X_{p}$ is lower than $X_{j}$, $X_{p}$ becomes the new candidate solution, and vice versa. The new candidate solution chosen in this stage is kept as the final solution for each iteration.

#### 2.2.4 Objective function

The objective function of the inversion process is to achieve a high fitness rate between the calculated response and the observed response of TDEM data. Fitness rate is associated with the misfit value, where a small misfit value shows a good fitness between the calculated response and the observed response of TDEM data. The misfit value is calculated using the Relative Root-Mean-Square Error (RRMSE) equation (16):

(16) $$E_{RRMSE}=\sqrt{\frac{1}{N}\sum_{J=1}^{N}{\left(\frac{\partial Bdt^{cal}-\partial Bdt^{obs}}{\partial Bdt^{obs}}\right)}^{2}}x100$$

With $\partial Bdt^{obs}$ and $\partial Bdt^{obs}$ are observed secondary derivative magnetic field and calculated secondary derivative magnetic field, respectively and *N* is a number of data.

## 3 Results

### 3.1 Synthetic data

**Fig. 2:**
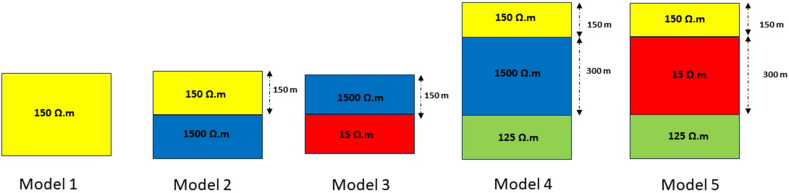
Illustration of synthetic models: Model 1 represents a homogeneous layer, Model 2 features two layers with the first layer being resistive and the second layer being more conductive, Model 3 presents two layers with the first layer being more conductive than the second layer, Model 4 showcases three layers with the second layer being more resistive than the others, and Model 5 displays three layers with the second layer being more conductive than the other layers.

To assess the algorithm's performance, various synthetic models were used (Fig. 2). These models include homogeneous models, two-layer models of two different types, and three-layer models of two different types. Parameters such as transmitter current, transmitter loop radius, and measurement time were defined to obtain TDEM data responses. For the transmitter current, a current of 2 A was used. The radius of the transmitter loop was set to 50 m. The measurement time interval ranged from 10^−6^ s – 1 s, with 30 sampling time points.

**Fig. 3:**
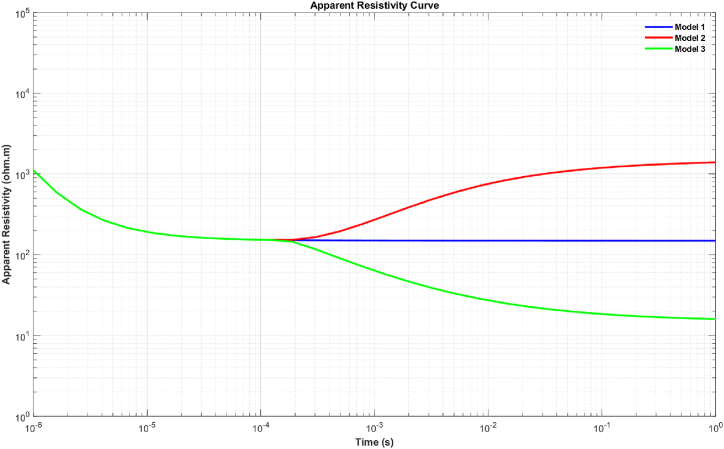
Late-time apparent resistivity for model 1, model 2, and model 3.

**Fig. 4:**
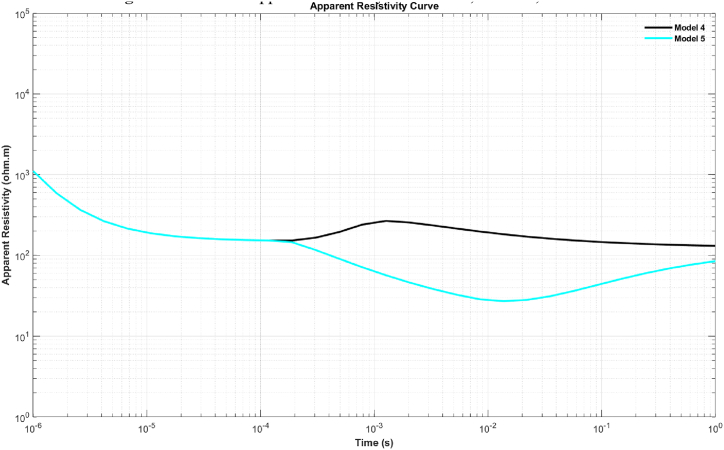
Late-time apparent resistivity for model 4 and model 5.

In the inversion of synthetic data, the late-time resistivity for each model was calculated using Equation (4). The visualization of each model is shown in Fig. 3, Fig. 4.

**Fig. 5:**
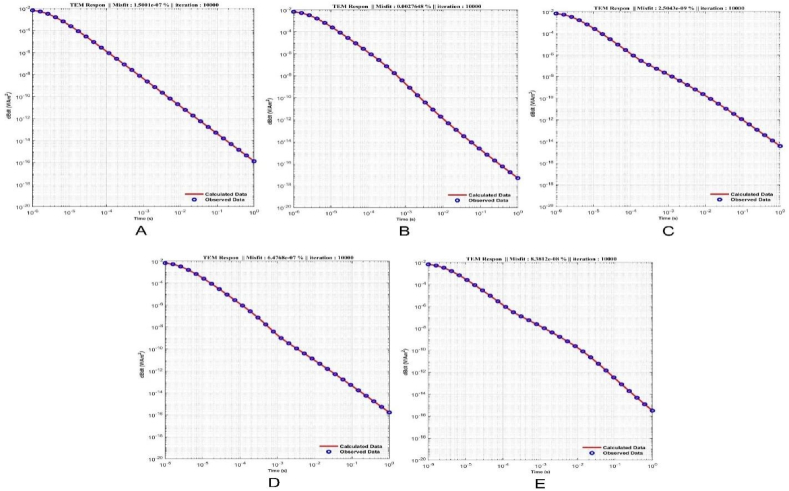
The response results of TDEM synthetic data and the fitting between synthetic and calculated synthetic data using the mSOS algorithm across several different layer models are as follows: A) Homogeneous Layer, B) Two Layers, C) Three Layers, D) Four Layers, and E) Six Layers.

**Fig. 6:**
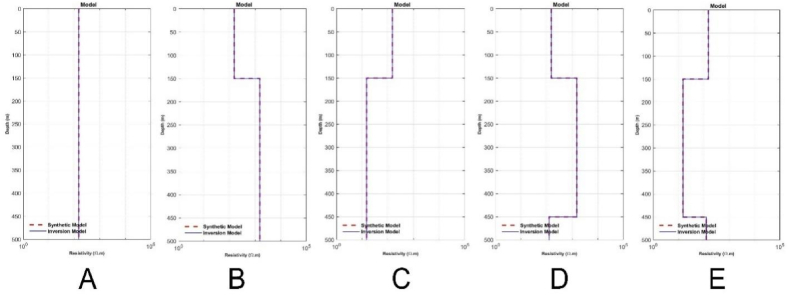
The model outcomes from the inversion of TDEM synthetic data using the mSOS algorithm across various layer models are as follows: A) Homogeneous Layer, B) Two Layers, C) Three Layers, D) Four Layers, and E) Six Layers.

To generate the initial random models for the inversion of synthetic data, the lower and upper boundaries for the resistivity and thickness parameters were set in the intervals of 1–2000 Ω m and 1–1000 m, respectively. The inversion parameters, including the number of iterations and the number of models, were set to 10,000 and 100, respectively. The results for each model are presented in Table 1, which includes the calculated parameters, the inversion process time, and the RRMSE. Fig. 5 (A-E) and Fig. 6 (A-E) sequentially present the TDEM data responses between measured and calculated data and the inversion models obtained through calculations for models 1 through 5, as described in Table 1. The results of the inversion models show consistent outcomes between the initial model and the model calculated with well-fitted measured and calculated data using the mSOS algorithm, as depicted in Table 1.

**Table 1:** Inverse results from synthetic data.

Inversion Results for Synthetic Model					
Parameter Model	Model 1	Model 2	Model 3	Model 4	Model 5
$\rho_{1}$ (Ω.m)	150	149.99	150	150	150
$\rho_{2}$ (Ω.m)	–	1500.03	15	1499.99	15
$\rho_{3}$ (Ω.m)	–	–	–	124.99	125
$h_{1}$ (m)	∞	149.99	149.99	149.99	149.99
$h_{2}$ (m)	∞	∞	∞	300	300
$h_{3}$ (m)	∞	∞	∞	∞	∞
Time (s)	955.45	761.96	732.95	1100.45	984.67
$E_{RRMS}$ (%)	1.50 $x\;10^{-7}$	2.76 $x\;10^{-3}$	2.50 $x\;10^{-9}$	6.48 $x\;10^{-7}$	8.38 $x\;10^{-8}$

To evaluate the performance of the algorithm in retrieving the parameter models, the error relative for each parameter model was calculated using Equation (17). The synthetic parameter models were compared with the calculated parameter models, and the results of the error relative calculations are shown in Table 2.

(17) $$Error\;Relatif\;\left(\%\right)=\left|\frac{Synthetic\;Parameter\;Model-Calculated\;Parameter\;Model}{Synthetic\;Parameter\;Model}\right|x100\%$$

**Table 2:** Error relative between synthetic model and calculated model.

Error Relative for Parameter Model (%)					
Parameter	Model 1	Model 2	Model 3	Model 4	Model 5
$\rho_{1}$ (Ω.m)	0.00 %	0.00 %	0.00 %	0.00 %	0.00 %
$\rho_{2}$ (Ω.m)	–	0.00 %	0.00 %	0.00 %	0.00 %
$\rho_{3}$ (Ω.m)	–	–	–	0.00 %	0.00 %
$h_{1}$ (m)	–		0.00 %	0.00 %	0.00 %
$h_{2}$ (m)	–	–	–	0.00 %	0.00 %
$h_{3}$ (m)	–	–	–	–	–

### 3.2 Field data

In the evaluation of field data, the algorithm was employed to execute inversion modeling utilizing Time Domain Electromagnetic (TDEM) data collected at the Volvi basin. The data specifically pertained to profile 3 at station 76 (TEM-76), situated to the northeast of Thessaloniki city in Northern Greece. The research, conducted by Widodo [11], aimed to investigate the near-surface structure. The measurements were performed using NT-20 and GDP-32 II transmitters, with the transmitter loop dimensions of 50 m × 50 m and receiver loop dimensions of 10 m × 10 m [11].

In the field data inversion process, various starting models with different numbers of layers were implemented. Specifically, the systematic consideration of models with two, three, four, and five layers was undertaken to thoroughly explore and assess the outcomes of the inversion process.The primary goal of this method was to ensure consistency in the number of layers within the field while considering the existing geological conditions. This consistency is gauged through fitting values between measured and calculated data, revealing a robust fit with low RRMSE values.

**Fig. 7:**
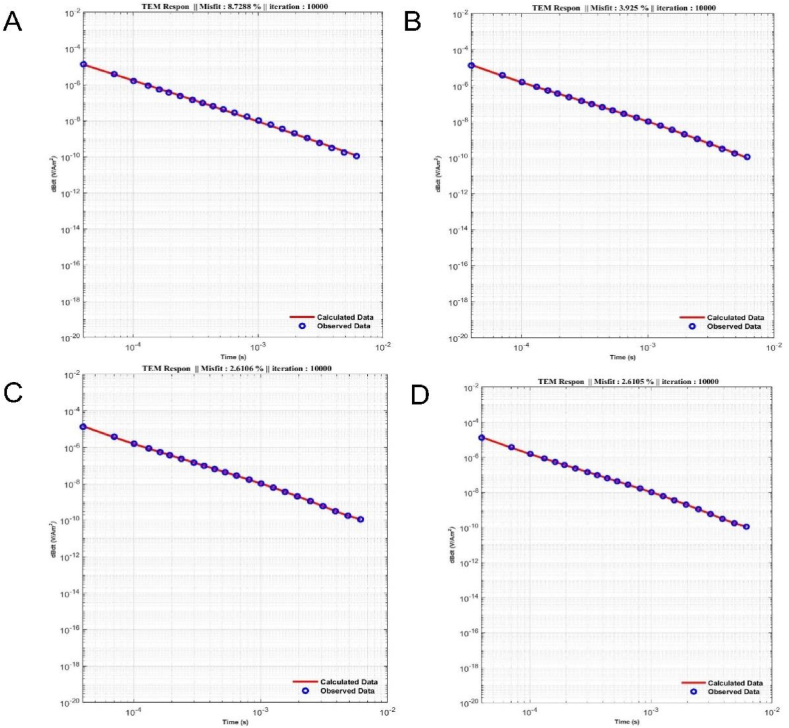
The outcomes of the TDEM field data response, along with the fitting between measured and calculated data using the mSOS algorithm, vary across different layer models, specifically: A) Homogeneous Layer, B) Two Layers, C) Three Layers, and D) Four Layers.

**Fig. 8:**
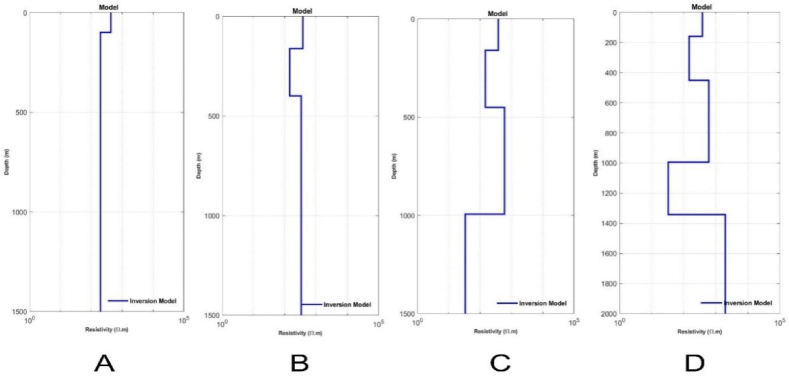
The inversion results of TDEM field data using the mSOS algorithm were obtained with different starting models employing varying numbers of layers. A) Starting model with two layers. B) Starting model with three layers. C) Starting model with four layers. D) Starting model with five layers.

Sequentially depicted inFig. 7 (A-D) and Fig. 8 (A-D), these illustrations highlight TDEM data responses, and the models derived from the inversion results for starting models of two, three, four, and five layers, detailed in Table 3. The fitting results in Table 3 highlight that the most favorable ERRMS value is associated with the starting model featuring five layers, recorded at 2.6105 % (see Fig. 7).

**Table 3:** Inverse results of field data.

Inversion Results for Field Data				
Parameter model	2 Layers Model	3 Layers Model	4 Layers Model	5 Layers Model
$\rho_{1}$ (Ω.m)	433.54	380.56	381.06	381.05
$\rho_{2}$ (Ω.m)	198.93	143.62	146.33	146.33
$\rho_{3}$ (Ω.m)	–	339.24	604.03	603.53
$\rho_{4}$ (Ω.m)	–	–	33.06	33.02
$\rho_{5}$ (Ω.m)	–	–	–	1999.96
$h_{1}$ (m)	99.03	161.34	159.63	159.63
$h_{2}$ (m)	∞	238.42	290.45	290.45
$h_{3}$ (m)	∞	∞	542.14	542.31
$h_{4}$ (m)	∞	∞	∞	350.52
$h_{5}$ (m)	∞	∞	∞	∞
Time (s)	410.38	470.81	513.88	541.19
$E_{RRMS}$ (%)	8.7288	3.925	2.6106	2.6105

Ensuring consistency in the number of layers within the field using starting models with different layer counts is critical for accurately deciding the actual number of layers present. This comprehensive process involves considering geological factors and addressing the ill-posed nature of model parameters to achieve the best fit, as elucidated by Jupp and Vozoff (1975) [12].

## 4 Discussion

The results of the inversion process using the mSOS algorithm for both synthetic data and field data prove its effectiveness in achieving satisfactory inversion results. For the synthetic data case, the algorithm was able to generate models with a high fitness rate, as showed by the small misfit values between the calculated and synthetic data. The relative error between the synthetic parameter models and the calculated models was also small, indicating a good agreement. Interestingly, the wide interval search space used in the random model generation did not significantly affect the inversion process. The algorithm was able to converge to a good solution with parameter values very close to or even similar to the true models.

In the field data case, the inversion process was conducted with similar parameter settings as the synthetic data case. Fig. 8 illustrates the inversion results of TDEM data using the mSOS algorithm with different starting models employing varying numbers of layers. Fig. 8 (A-D) presents the inversion outcomes of field data processed with starting models of two layers, three layers, four layers, and five layers consecutively. The resistivity patterns for each layer were consistent, and even though the thickness of the first layer differed significantly from the other models, the remaining three models showed insignificant differences in their parameter values.

**Fig. 9:**
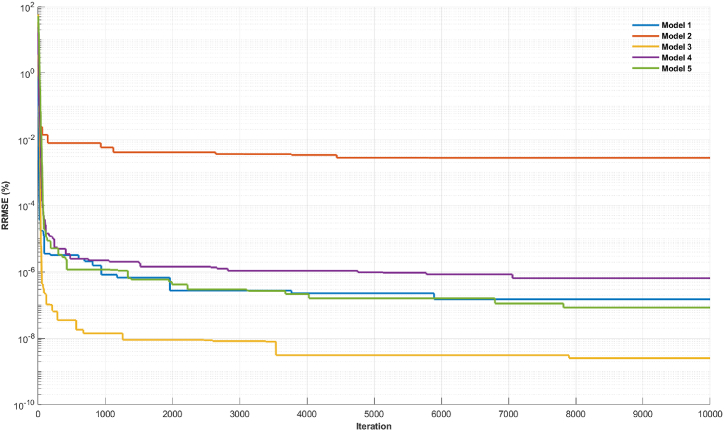
Misfit graphic for synthetic data case.

**Fig. 10:**
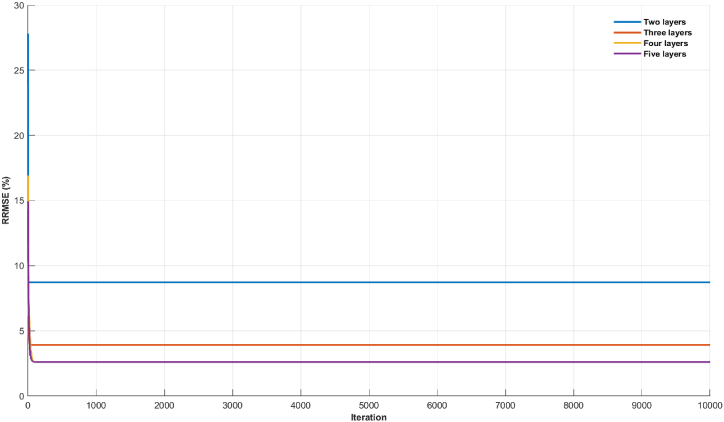
Misfit graphic for field data case.

In Fig. 9, Fig. 10, the diminishing misfit values with an increase in the number of layers suggest the existence of multiple layers with distinct lithologies in the subsurface. A comparison between the visualizations of misfit values in synthetic data modeling and field data modeling reveals a slight variance in the behavior of the objective function curve. In the case of synthetic data, there was a likelihood for the algorithm to attain an improved misfit value at a relatively high number of iterations, approximately 5000 to 8000, before reaching a plateau in the objective function curve (Fig. 9). Conversely, the field data modeling exhibited a faster convergence of the objective function, requiring fewer iterations (<100) (Fig. 10). This dissimilarity can be attributed to the inherent complexity of field data, where noise introduces scattering, potentially impacting the algorithm's calculation process. Consequently, achieving convergence in the inversion process for field data necessitated fewer iterations compared to synthetic data.

**Fig. 11:**
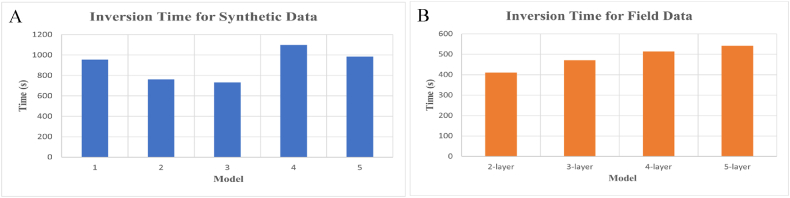
Inversion time for (A) synthetic data and (B) field data.

The number of iterations was set to 10,000 for all cases to test the speed of the algorithm and ensure convergence of the objective function, as indicated by the insignificant change in the misfit value for each iteration. The inversion time for all cases, as shown in Fig. 11, was relatively fast, taking approximately less than 19 min to complete. Fig. 11 (A) depicts the time required during the inversion process for synthetic data. The shortest and longest durations for the inversion process of synthetic data are observed consecutively for models 3 and 4. Meanwhile, models 2 and 5 exhibit the fastest and slowest time comparisons for field data processing, as illustrated in Fig. 11 (B). It's worth noting that the inversion time can be influenced by hardware performance, and running other programs simultaneously may slow down the computation process. Overall, the mSOS algorithm proves excellent performance in terms of accuracy and computation time for TDEM data inversion.

Comparison between the Levenberg Marquardt and mSOS Algorithm's.

To assess the consistency and advantages of the mSOS algorithm for processing TDEM data, a comparison was conducted with the Levenberg-Marquardt (L-M) algorithm [13]. The L-M algorithm has been widely used for TDEM data processing and has shown relevant results in TDEM data inversion models [5,11,14]. For the comparative testing of the L-M algorithm with mSOS, field data from profile 3 station 83 (TEM-83) were employed, referring to Widodo et al.'s research [11].

In this case, deciding the number of subsurface layers and assessing the reasonableness of inversion results is a multidisciplinary process. It involves geological knowledge consideration, model selection criteria application, cross-validation techniques use, well data or borehole information incorporation, and complementary geophysical imaging techniques integration. By combining these approaches, a more robust assessment of inversion results can be achieved, leading to a better understanding of subsurface structures. The inversion parameter approach conducted involved using starting models of two layers, three layers, four layers, and five layers for both the L-M and mSOS algorithms, with an iteration process of 50 times.

**Fig. 12:**
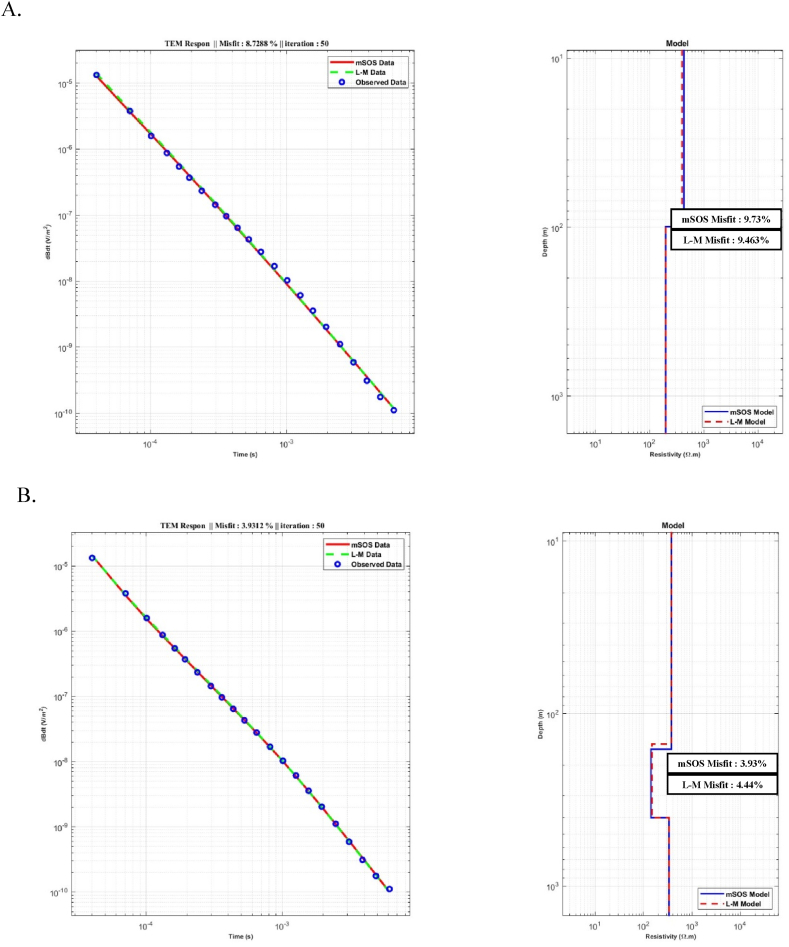

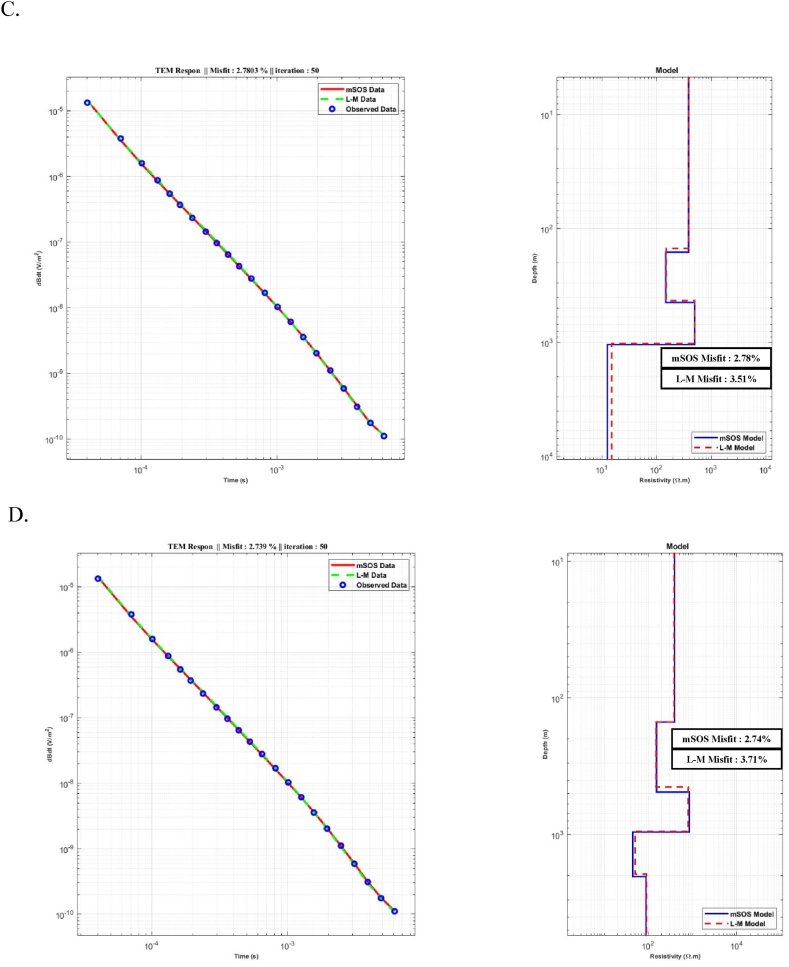
A comparison between the L-M and mSOS algorithms for inversion models was conducted using different starting models with varying numbers of layers. A) Starting model with two layers. B) Starting model with three layers. C) Starting model with four layers. D) Starting model with five layers.

Fig. 12 illustrates the comparison results between the inversion models produced by the L-M and mSOS algorithms. In general, the inversion models generated by the mSOS algorithm exhibited consistent and acceptable results across various numbers of layer models compared to the L-M algorithm. Notably, the misfit values showed a sequential decrease with an increasing number of layers $E_{RRMS}$ (%). Starting at 9.7 % for the 2-layer model (Fig. 12 (A), the misfit values progressively reduced to 3.93 % for the 3-layer model and further to 2.78 % (Fig. 12 (B) for the 4 and 5-layer models, with an $E_{RRMS}$ (%) of 2.74 % Fig. 12 (C) and (D). On the other hand, L-M showed a better RRMS error result for the starting model using two layers, which was 9.46 %. However, for other models, it was higher compared to the mSOS algorithm. The data comparison of $E_{RRMS}$ (%) from field data for the inversion models of the L-M and mSOS algorithms can be seen in Table 4.

**Table 4:** Comparison between the L-M and mSOS algorithm's for inversion models.

Inversion Results for Field Data				
Algorithm's	2 Layers Model	3 Layers Model	4 Layers Model	5 Layers Model
Levenberg Marquardt $E_{RRMS}$ (%)	9.46	4.44	3.51	3.71
mSOS $E_{RRMS}$ (%)	9.73	3.93	2.78	2.74

In the comparison between the L-M and mSOS algorithms for processing TDEM field data, the results indicate consistent outcomes in terms of the number of layers and layer thicknesses. However, the mSOS algorithm achieves a better fitting between the measured and calculated data compared to the L-M algorithm. This specific outcome can be influenced by several factors, including.

● Local Minima: L-M can get trapped in local minima, particularly when the initial guess is far from the global minimum. This may lead to suboptimal solutions with higher RRMS errors. In contrast, mSOS explores multiple starting points, increasing the chances of finding the global minimum.
● Non-Linearity Handling: TDEM data inversion involves complex, non-linear relationships between observed data and subsurface properties. L-M employs linearization techniques that might struggle to accurately represent these non-linearities, resulting in higher RRMS errors. mSOS is better equipped for non-linear problems.
● Noise Sensitivity: TDEM data often contain noise and uncertainties. L-M can be sensitive to noise, leading to suboptimal convergence and higher RRMS errors. mSOS iterative and averaging approach can mitigate noise effects, resulting in more accurate solutions.
● Model Complexity: In cases with intricate subsurface models, including numerous layers or complex structures, L-M may have difficulty handling the complexity, leading to higher RRMS errors. mSOS algorithm ability to explore various model complexities can produce more accurate results.
● Global Optimization: L-M's convergence heavily relies on the initial guess and can struggle to explore different regions effectively. mSOS, with its diverse starting points, enhances global optimization capabilities, reducing the likelihood of getting stuck in local minima.
● Diversity of Solutions: TDEM data inversion can yield multiple valid solutions due to data limitations or geological uncertainties. L-M may converge to one of these solutions, potentially resulting in higher RRMS errors if it's not the most suitable solution. mSOS algorithm diverse starting points explore a broader solution space.
● Algorithm Sensitivity: L-M's performance can be sensitive to optimization parameters like step size, regularization, and termination criteria. Poor parameter choices can lead to higher RRMS errors. mSOS Algorithm broader exploration approach may be more robust to parameter variations.

These specific reasons highlight how L-M's characteristics, including its susceptibility to local minima, challenges with non-linear problems, and sensitivity to noise, can contribute to higher RRMS errors compared to the mSOS algorithm when applied to TDEM data inversion. This trend suggests the mSOS algorithm's ability to effectively capture subsurface structures and enhance the overall accuracy of the inversion process.

## 5 Conclusion

In summary, the implementation of the mSOS algorithm for central-loop TDEMdata inversion has yielded significant advancements and promising outcomes for both synthetic and field data cases.

For the synthetic data case, the mSOS algorithm successfully retrieved the synthetic model with remarkable accuracy, showing only a small relative error compared to the original synthetic model. The calculated response closely matched the observed data, as shown by the exceptionally low misfit values. In fact, the overall misfit values for all models approached an impressive 0 %, showing a near-perfect fit between the calculated and observed data.

Moving on to the field data case, the inversion models generated by the mSOS algorithm highlighted consistent and acceptable results across various numbers of layer models. Notably, the misfit values proved a sequential decrease with an increasing number of layers. This trend suggests the algorithm's ability to effectively capture the subsurface structures and improve the overall accuracy of the inversion process.

In addition to the impressive accuracy achieved, the mSOS algorithm exhibited a notable advantage in terms of computational efficiency. The inversion process proved to be remarkably fast, with the completion of 10,000 iterations requiring less than 19 min. This time efficiency makes the algorithm particularly advantageous for practical applications, where timely analysis and decision-making are crucial.

In comparison to the L-M inversion method, the mSOS algorithm consistently produces results that align with the models generated by the L-M algorithm and achieves a better fit between the measured and observed data than the L-M algorithm.

Overall, this study contributes to the field by introducing and highlighting the innovative mSOS algorithm for TDEM data inversion. The algorithm's ability to accurately retrieve models, closely match observed data, and exhibit computational efficiency is highly promising. These findings prove the algorithm's potential to revolutionize TDEM data inversion studies and enhance our understanding of subsurface structures. Further research and application of the mSOS algorithm are warranted to explore its full capabilities and address potential challenges in different geological scenarios.

## Funding

This research was funded by the Ministry of Research, Technology, and Higher Education (RISTEKDIKTI) of Indonesia. The authors also gratefully acknowledge a research grant from the Program of Research, Community Service, and Innovation (10.13039/100020300LPPM) of the Institute Technology of Bandung (10.13039/501100015689ITB) and National Research and Innovation Agency/Badan Riset Inovasi Nasional (BRIN).

## Data availability

Data included in the article.

## CRediT authorship contribution statement

**Widodo:** Conceptualization, Data curation, Formal analysis, Funding acquisition, Investigation, Methodology, Project administration, Resources, Software, Supervision, Validation, Visualization, Writing – original draft, Writing – review & editing.

## Declaration of competing interest

The author declare that they have no known competing financial interests or personal relationships that could have appeared to influence the work reported in this paper.
